# Subjective memory complaints in the elderly may be related to factors other than cognitive deficit

**DOI:** 10.1590/S1980-57642010DN40100009

**Published:** 2010

**Authors:** Ana Cristina Procópio de Oliveira Aguiar, Miriam Ikeda Ribeiro, Alessandro Ferrari Jacinto

**Affiliations:** 1 MSc, Psychologist at the Residencial Israelita Albert Einstein.; 2 RN, Manager at the Residencial Israelita Albert Einstein.; 3 MD, PhD, Geriatrician at the Residencial Israelita Albert Einstein, Member of the Behavioral and Cognitive Neurology Group of the Neurology Department, Faculty of Medicine, University of São Paulo.

**Keywords:** elderly, subjective memory complaint, mild cognitive impairment

## Abstract

Prevalence of subjective memory complaints (SMC) in elderly community dwellers (ECD) ranges from 25 to 50%. Some controversy exists over the association between SMC and cognitive status.

**Objective:**

We investigated whether a correlation in cognitive performance existed between two different groups according to SMC.

**Methods:**

The Mini Mental State Examination (MMSE) and Dementia Rating Scale-Mattis (DRS-Mattis) were applied to two groups: ECD (n=14) with SMC and residents (n=14) of a long-term care facility (LTCF) without SMC.

**Results:**

The median age in the ECD group was 81.0 years, and in the LTCF group was 75.0 years. There was a statistically significant difference (p=0.048) between these groups regarding age. Concerning schooling (1-8 or ≥9 years), there was no statistically significant difference between the groups (p=0.638). No statistically significant difference between the two groups was found for scores on the cognitive tests.

**Conclusion:**

SMC might be related to extrinsic factors other than insipient cognitive decline.

## Abstract (pt)

A prevalencia de queixa subjetiva de memoria (QSM) em idosos da comunidade (IRC) varia de 25-50%. Controversias existem a respeito da associacao entre QSM e cognição.

**Objetivos:**

Foi investigado se ha correlacao entre QSM e declinio cognitivo em dois diferentes grupos.

**Metodo:**

Foi aplicado o Mini Exame do Estado Mental (MEEM) e a Escala de Avaliacao de Demencia Mattis (DRS-Mattis) em dois grupos de idosos: IRC (n=14) que apresentaram QSM e residentes (n=14) de uma instituicao de longa permanencia (ILP) sem QSM.

**Resultados:**

A mediana de idade do grupo "IRC" foi de 81,0 anos e a do grupo "ILP" foi de 75,0. Houve diferenca estatisticamente significante (p=0,048) entre eles, considerando a idade. Nao houve esta diferenca (p=0,638) na distribuicao dos individuos quando analisada a escolaridade (1-8 ou ≥9 anos). Considerando-se os escores dos instrumentos cognitivos, nao houve diferenca estatisticamente significante entre grupos.

**Conclusões:**

QSM pode estar relacionada a outros fatores em idosos, e nao somente a presenca de declinio cognitivo.

The prevalence of subjective memory complaints (SMC) in elderly community dwellers (ECD) ranges from 25 to 50%.^1^ Based on recent studies the correlation between these complaints and cognitive performance remains controversial.^2,3^

There is some evidence in the medical literature suggesting that SMC may be indicative of pathological cognitive deficit such as mild cognitive impairment (MCI) or dementia.^4,5^ Regarding MCI, SMC itself is a valuable predictor in the diagnostic process. Moreover, SMC in patients with MCI may represent an increased risk for developing dementia.^1,6-8^

Some authors suggest that ECD with cognitive complaints may reflect co-morbidities other than dementia, such as mood or depression.^3,9-11^ Similar findings have not been replicated with the elderly living in a Long Term Care Facility (LTCF), perhaps because the two populations live in highly heterogeneous settings. In LTCF, there are rules to be followed and goals to be achieved based on interdisciplinary protocols. Besides the social-psychological and biological conditions differentiating this population, individuals living in LTCF receive daily care that in most cases is passively accepted. This scenario may directly interfere with the relationship between this population and the environment, depriving these individuals of exerting a decision-making capacity that would require their cognitive capacity.^12,13^

In this study, the cognitive status of two groups of elderly people living in the two settings outlined was compared. While one group comprised individuals living in a LTCF, the other consisted of community dwellers. These two groups comprised Jewish elderly individuals born in Europe but living in Brazil since their youth.

The study was performed in the metropolitan area of the city of São Paulo, Brazil, in December of 2008.

## Methods

Twenty-eight elderly individuals were analyzed regarding their cognitive status. Participants were routinely followed by the Geriatrics Staff of Albert Einstein Jewish Hospital - Vila Mariana Unit. Fourteen participants were ECD outpatients and 14 lived in an LTCF. The global health assessment protocol applied to these elders contained tests for cognitive status evaluation including the MMSE and Mattis DRS besides a question about memory complaints (“How is your memory?”). In this study, MMSE and DRS-Mattis scores and the presence of SMC were the only variables of the protocol taken into account in the analysis of the correlation between SMC and objective cognitive performance.

DSM-IV diagnostic criteria for dementia were used in order to classify the individuals as demented or non-demented.

Initially, descriptive statistics were used to obtain median and interquartile ranges of some variables. To verify for a normal distribution of test results, the Kolmogorov-Smirnov analysis was used. The median scores for the two groups were compared using the Mann-Whitney test. Qualitative variables between the groups were compared by the Chi-square test. Fisher’s test was used when the assumptions of the Chi-square test were not confirmed.

The Ethics Committee of the Research and Teaching Institute of the Albert Einstein Jewish Hospital approved this study. All the participants signed a consent form before taking part in the study.

## Results

In the ECD group, 70.6% were female and the median age was 81.0 (78.0-86.0) years. In the LTCF group, 53.8% were female and the median age was 75.0 (71.5-83.5) years. There was a statistically significant difference (p=0.048) between the two groups regarding age.

In the ECD group, 8 elders (47.1%) had 1-8 years of schooling and 9 (52.9%) had 9 or more years of schooling. In the LTCF group, 5 elders (38.5%) had 1-8 years of schooling and 8 (61.5%) had 9 or more years of schooling.

There was a statistically significant difference in median age between the two groups (p=0.048). Regarding schooling, no statistically significant difference was observed between the 2 groups (p=0.638).

Regarding SMC, all the ECD had memory complaints, whereas none of the LTCF elders had SMC.

The median scores on the MMSE were 25.0 (23.0-29.0) in the LTCF group and 28.5 (24.5-29.75) in the ECD group (Table 1). The median scores on the Mattis DRS were 127.0 (113.5-135.0) and 122.0 (115.0-133.0) in the LTCF and ECD groups, respectively (Table 2). No statistically significant difference was found between the two groups in MMSE (p=0.268) and DRS-Mattis scores (p=0.650).

**Table 1:** Cognitive Performance of LTCF and ECD groups on the MMSE.

MMSE	Median (IQR)		p*
	**LTCF**	**ECD**	
Orientation	10.00(9.00-10.00)	10.00(9.00-10.00)	0.983
Immediate recall	3.00(3.00-3.00)	3.00(3.00-3.00)	0.746
Attention and calculation	5.00(3.00-5.00)	5.00(4.00-5.00)	0.650
Delayed recall	2.00(0.25-2.00)	2.00(1.00-3.00)	0.397
Language	8.00(7.00-8.00)	8.00(8.00-8.00)	0.288
Copying	1.00(1.00-1.00)	1.00(1.00-1.00)	0.975
Total score	25.00(23.00-29.00)	28.50(24.50-29.75)	0.268

* Mann-Whitney test; LTCF, Long Term Care Facility; ECD, Elderly Community Dwellers; MMSE, Mini-Mental State Examination; IQR, Inter-quartile Range.

**Table 2:** Cognitive Performance of LTCF and ECD groups on the Mattis DRS.

Mattis DRS	Median (IQR)		p*
	**LTCF**	**ECD**	
Attention	34.00(33.00-35.50)	34.00(32.00-35.00)	0.235
Initiation/ Perseveration	33.00(27.50-36.00)	30.00(26.00-33.00)	0.156
Construction	6.00(4.50-6.00)	6.00(6.00-6.00)	0.316
Conceptualization	37.00(24.50-38.50)	37.00(32.00-39.00)	0.964
Memory	19.00(15.50-22.50)	19.00(17.00-22.00)	0.751
Total Score	127.00(113.50-135.00)	122.00(115.00-133.00)	0.650

* Mann-Whitney test; LTCF, Long Term Care Facility; ECD, Elderly Community Dwellers; Mattis DRS, Mattis Dementia Rating Scale; IQR, Interquartile Range.

According to DSM-IV dementia diagnostic criteria, none of the subjects of this study were classified as demented.

## Discussion

Individuals with a Jewish background belong to a very distinct cultural environment compared to that of other Brazilians. In the Jewish community, it is very important to be involved in intellectual activities throughout life. A link has been shown between leisure activities and lower incidence of cognitive decline.^14-17^ This could be the reason behind the absence of dementia diagnoses in our study, although the number of subjects in this study was not large.

The different prevalence of SMC found between the two groups is noteworthy. Elders living in LTCF receive daily interdisciplinary assessment that might be conducive to a passive way of life, allowing them to be unconcerned about everyday stressful decisions that are otherwise required to be taken by individuals.^12,13^ ECD elders however, require greater awareness of their daily life activities such as taking medication, cooking, cleaning, and self-hygiene. Therefore, the need to exercise these daily skills may put these individuals in closer touch with their abilities to perform everyday tasks, which in turn allows them to identify difficulties in carrying out these tasks. This may explain the increased prevalence of SMC in the LTCF population. Alternatively, because ECD do not receive specialized care for their needs, it is possible that reporting of memory complaints could be an indirect way of either asking for help or seeking greater attention from health care professionals. We found no other studies in the literature comparing these two types of elder population with regard to SMC.

Although LTCF residents do not need to worry about self-care, they have more frequent leisure and intellectual activities than do elders living in the community. For instance, the studied LTCF runs a program called “Day Center” in which both ECD and LTCF residents meet twice a week to take part in a host of activities such as nutrition classes, cognitive stimulation, and physical activities. Results of a quality of life questionnaire (SF-36)^18^ applied to these Day Center attendees revealed that community dwellers had more complaints related to social life (unpublished data). No daily assessments of functional activities or mood were performed in our subjects; this represents a limitation of the present study.

People age differently and their life experiences contribute directly to this aging process. The growth in the geriatric population should provide a better understanding of the different issues involved in aging.^19^ Diagnosis of cognitive impairment must be determined carefully and as part of this process, memory complaints should be taken into consideration and evaluated in more depth.

To conclude, SMC is an important element in dementia diagnosis but should be taken into consideration in the context of other individual characteristics such as the elder’s living conditions.
